# In vivo MRI assessment of permanent middle cerebral artery occlusion by electrocoagulation: pitfalls of procedure

**DOI:** 10.1186/2040-7378-2-4

**Published:** 2010-02-04

**Authors:** Fabien Chauveau, Samir Moucharrafie, Marlène Wiart, Jean-Christophe Brisset, Yves Berthezène, Norbert Nighoghossian, Tae-Hee Cho

**Affiliations:** 1 CREATIS, CNRS UMR 5220, INSERM U 630, Université Claude Bernard Lyon I, France; 2 Cerebrovascular Unit, Hôpital Neurologique Pierre Wertheimer, Lyon, France

## Abstract

Permanent middle cerebral artery (MCA) occlusion (pMCAO) by electrocoagulation is a commonly used model but with potential traumatic lesions. Early MRI monitoring may assess pMCAO for non-specific brain damage. The surgical steps of pMCAO were evaluated for traumatic cerebral injury in 22 Swiss mice using diffusion and T2-weighted MRI (7T) performed within 1 h and 24 h after surgery. Temporal muscle cauterization without MCA occlusion produced an early T2 hyperintensity mimicking an infarct. No lesion was visible after temporal muscle incision or craniotomy. Early MRI monitoring is useful to identify non-specific brain injury that could hamper neuroprotective drugs assessment.

## Introduction

Investigating cerebral ischemia requires animal models relevant to human stroke. A precise knowledge of the strengths and shortcomings of available models is mandatory for effective research in neuroprotection [1,2]. Initially described in the rat [3] and subsequently adapted in mice [4], permanent middle cerebral artery (MCA) occlusion (pMCAO) by electrocoagulation is a widely used model of focal ischemia. Invasive surgical procedures are required: temporal muscle dissection, in some cases by electrical cauterization [5,6], subtemporal craniotomy and MCA electrocoagulation. This model, by interrupting blood flow at the level of the parietal cerebral artery branch of the MCA (distal occlusion), has the advantage of producing smaller, cortical-restricted, more reproducible and better-tolerated infarcts compared to suture MCAO, which endoluminal occluder is situated in the internal artery, at the birth of the MCA (proximal occlusion) and gives rise to extensive cortico-striatal infarcts [7,8]. It may however induce traumatic brain damage.

To our knowledge, sham-operated animals have only been studied using immunohistology at the subacute stage of cerebral ischemia. Early monitoring with magnetic resonance imaging (MRI) may facilitate in vivo identification of traumatic brain injury during pMCAO.

## Materials and methods

### Animals and surgical procedure

We designed an analytic appraisal of pMCAO procedure which included two methods of temporal muscle dissection: cauterization and blade incision. After intraperitoneal anesthesia with 12 mg/Kg xylazine and 90 mg/Kg ketamine, 22 male Swiss mice (28-30 g, Charles River, France) were allotted as follows:

• group A (n = 6): temporal muscle cauterization without craniotomy nor MCA occlusion;

• group B (n = 4): temporal muscle incision without craniotomy nor MCA occlusion;

• group C (n = 4): temporal muscle incision followed by craniotomy without MCA occlusion;

• group D (n = 6): temporal muscle incision followed by craniotomy and MCA electrocoagulation;

• group E (n = 2): temporal muscle cauterization followed by craniotomy and MCA electrocoagulation.

Craniotomy and MCA electrocoagulation were performed as previously described [9]. Whatever the group be, surgery was performed in less than 15 min.

### MRI

MRI experiments were performed on a 7T/12 cm magnet (Bruker BioSpin GmbH, Ettlingen, Germany) using a 72 mm inner diameter birdcage for RF transmission and a 15 mm diameter surface coil for reception. T_2_-weighted images (T_2_WI) were acquired using a RARE sequence with TE/TR = 75/3000 ms. Diffusion weighted spin-echo images (DWI) were acquired with a TE/TR = 14/2000 ms using 2 b-values (139 and 1061 s/mm^2^). Apparent diffusion coefficients (ADC, in mm^2^/s) were calculated by fitting a monoexponential model function on a pixel-by-pixel basis. The field of view was 20 × 20 mm^2 ^and slice thickness 1.0 mm. For T_2_WI, 15 slices were acquired with 256 × 256 matrix and for DWI, 7 slices with 128 × 128 matrix. Mice anesthesia was maintained during the MRI with isofluorane (1.5% in air). Body temperature was kept at 37 ± 1°C with an integrated heating system, and a pressure probe monitored mice respiration. MRI was started immediately following the end of surgical procedure and all MR acquisitions were performed between 30 min and 60 min after the start of surgery. MRI was repeated on day 1.

## Results

**Figure 1:**
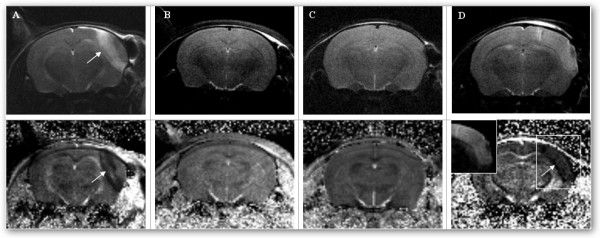
**MRI within one hour of surgery: T_2_WI (upper row) and ADC map (lower row)**. A: temporal muscle cauterization alone (group A). Note the lesion with early T_2_WI hyperintensity and reduced ADC (arrows). B: temporal muscle incision alone (group B). C: temporal muscle incision and craniotomy (group C). D: temporal muscle incision, craniotomy and MCA electrocoagulation. Note the ischemic lesion with low ADC (arrow) and normal T_2_WI with a limited superficial traumatic lesion (arrow).

**Figure 2:**
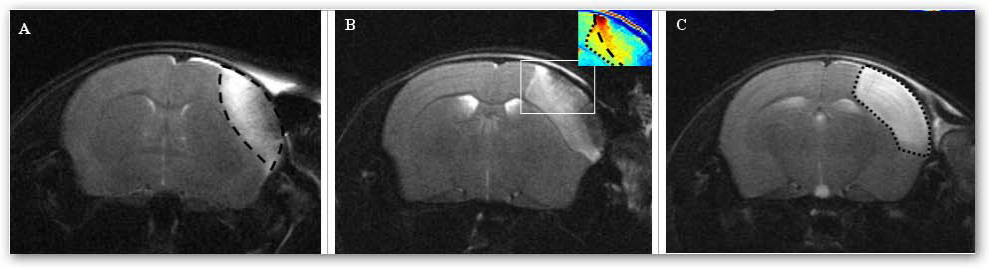
**Impact of temporal muscle cauterization on pMCAO with T_2_WI**. A: temporal muscle cauterization alone (group A) showing day 0 traumatic lesion (interrupted lines). B: temporal muscle cauterization, craniotomy and MCA electrocoagulation (group E). Color-coded magnification shows the boundaries of the ischemic lesion (dotted line) and traumatic lesion (interrupted lines) 6 h after pMCAO. C: same mice at day 1 showing the limits of the final lesion (dotted line).

Temporal muscle cauterization in the absence of MCA electrocoagulation (group A) consistently produced an extensive lesion across the frontoparietal cortex (n = 6/6). These lesions appeared hyperintense on T_2_WI within the first hour after surgery, with an early mass effect and reduced ADC (Figure 1A). Temporal muscle incision and craniotomy in the absence of MCA electrocoagulation (group B and C, respectively) caused no visible brain injury on day 0 or day 1 MRI (n = 4/4 in groups B and C, Figure 1B and 1C). MCA electrocoagulation (group D) induced an ischemic lesion in the frontoparietal cortex with reduced ADC on day 0 (n = 6/6). These lesions were not visible on T_2_WI within 1 h post-surgery (Figure 1D). No bleeding was noted during the surgery, but MCA electrocoagulation occasionally produced a small superficial traumatic lesion (n = 2/6 in group D, figure 1D). When pMCAO was performed using temporal muscle cauterization (group E), the lesion appeared as a superimposition of the almond-shaped cauterization lesion and of the MCA-territory bounded ischemic lesion (n = 2/2, Figure 2). Infarcts from group A, D and E were clearly delineated on day 1 T_2_WI.

## Discussion

Our results showed that temporal muscle cauterization in the absence of ischemic stimulus produced a hyperintense area on T_2_WI highly suggestive of traumatic injury. Indeed, T_2 _increase is not expected in ischemic lesions within the first hours of occlusion in adults, although early T_2 _increase has been reported in neonates after MCA electrocoagulation [10] or hypoxia-ischaemia [11]. Early mass effect is uncommon after ischaemia in the adult rat [12] or mouse [13]. Acute ADC decrease is not specific of ischemic insult and has been described in both experimental and human traumatic brain injury [14,15]. Signal suggestive of intra- or extra-cranial surgery-related bleeding (T2 hypointensity) would have been ascertained using T2*WI, but were not noted. No traumatic injury was observed after temporal muscle incision. Accordingly, incision should be preferred over cauterization for temporal muscle dissection.

The MRI appearance of lesions resulting from both temporal muscle cauterization (traumatic damage) and occlusion (ischemic damage) may mimic an infarction, especially if imaging is done at later time points. Histopathological analyses are usually performed after a delay of 24-72 h, when traumatic and ischemic damage may not be discernable, while early histological examination of intracerebral coagulation necrosis would be required to ascertain the thermal origin of traumatic damage induced by muscle cauterization.

In the last decade, high resolution MRI has become a valuable tool for monitoring tissue damage in rodent models of cerebral ischemia. Early MRI monitoring may help to identify non-specific brain injury that could hamper neuroprotective drugs assessment.

## Competing interests

The authors declare that they have no competing interests.

## Authors' contributions

FC carried out the MRI experiments, participated in the design of the study and helped to draft the manuscript. SM carried out surgery. MW designed and optimized the MRI acquisition protocol, and helped to draft the manuscript. JCB participated in the MRI experiments. YB and NN conceived the study, and participated in its design and coordination. THC conceived the study, participated in its design, performed image analysis, and drafted the manuscript. All authors read and approved the final manuscript.
